# Comprehensive analyses of biological function and tumor microenvironment with cuproptosis regulators and construction of a cuproptosis-related scoring system in thyroid cancer based on bioinformatics and experimental validation

**DOI:** 10.3389/fgene.2026.1735093

**Published:** 2026-02-27

**Authors:** Shitong Su, Jiaqi Han, Zijian Liu, Kun Tian

**Affiliations:** 1 Department of Pathology, West China Second University Hospital, Sichuan University, Chengdu, China; 2 Key Laboratory of Birth Defects and Related Diseases of Women and Children (Sichuan University), Ministry of Education, Chengdu, China; 3 Department of Radiation Oncology and Department of Head and Neck Oncology, Cancer Center, West China Hospital, Sichuan University, Chengdu, China; 4 Department of Andrology/Sichuan Human Sperm Bank, West China Second University Hospital, Sichuan University, Chengdu, China

**Keywords:** cuproptosis, Fdx1, thyroid cancer, tumor microenvironment, WGCNA

## Abstract

**Background:**

The role of copper-induced cell death, termed cuproptosis, has been demonstrated recently. Nonetheless, the potential biological function of cuproptosis regulators in thyroid cancer (THCA) remains unknown.

**Method:**

We analyzed the expression levels and prognostic values of cuproptosis regulators in THCA. We used weighted gene co-expression network analysis (WGCNA) and single-sample gene set enrichment analysis (ssGSEA) to perform biological function analysis. We used a sliding windows sequential forward feature selection (SWSFS) method to construct a cuproptosis-related score (RS) to predict progression- and disease-free survival probability. We validated the expression level of cuproptosis-related genes and explored the biological function of FDX1.

**Results:**

FDX1 was a protective factor for THCA and possessed a higher expression level in thyroid cancer. WGCNA and ssGSEA analysis showed that several pathways, such as protein secretion, oxidative phosphorylation, MYC, MTORC1, DNA repair, and adipogenesis, were highly positively correlated with cuproptosis regulators. In contrast, some immune-related pathways, such as interferon response and inflammatory pathways, were negatively correlated. We selected intersection genes by correlation analysis between the expression level of FDX1 and the prediction inhibitory concentration (IC50). Stratified analysis and nomogram were also employed to verify the validity and accessibility of the signature. Correlation analysis suggested that FDX1 expression was associated with immune cell infiltration patterns in THCA based on *in silico* estimations. Using qRT-PCR, we found that the expression level of MAP1LC3A and RBPMS2 were higher in normal thyroid tissues, and the GINM1 was higher in THCA. Colony forming and Cell Counting kit-8 assays verified that FDX1 might not affect cell growth. And by down-regulating FDX1, we found that the expression level of genes involved in cuproptosis RS might be affected by FDX1 in THCA.

**Conclusion:**

Overall, our study identified a novel cuproptosis-based predictive model, and we demonstrated that cuproptosis is a promising therapeutic method for THCA, which enhances our understanding of the cuproptosis-related genes and provides valuable insights into the clinical treatment and molecular mechanisms of THCA.

## 1 Introduction

Thyroid cancer (THCA) is the most common malignant tumor of the endocrine system, and its incidence (>3% per year) continues to increase year by year (James et al., 2018; Siegel et al., 2022). Up to date, the THCA has risen to the seventh most common malignant tumor among American women (Siegel et al., 2022), the global incidence is 10.2 per 100,000, and the disease accounts for 5.1% of all cancers (Bray et al., 2018). Among Chinese women, THCA has become the most commonly diagnosed cancer in young women (age <30) (Chen et al., 2016). Papillary thyroid carcinoma (PTC) is the most common type of THCA, accounting for more than 80% of all cases, and only 10% of patients might experience disease recurrence or metastasis (Kitahara and Sosa, 2016; Ito et al., 2018). However, poorly differentiated thyroid cancer (PDTC) and anaplastic thyroid cancer (ATC) might be more aggressive and fatal (Cabanillas et al., 2015). Although most PTC display indolent biological characteristics, most PTC has a relatively better prognosis after surgery and ^131^I treatment than other THCA subtypes (Xie et al., 2020). However, the recurrence and metastasis of certain PTC patients still obstruct clinical management and survival. Finding alternative treatment methods and constructing a valuable prognostic model might improve the survival of patients with THCA.

Regulated cell death (RCD) is the primary mechanism for eliminating damaged, infected, or redundant cells, which could play an essential role in tumorigenesis (Sauler et al., 2019; Tang et al., 2019). Several studies have reported that genes involved in RCD, such as autophagy (Hu et al., 2020), ferroptosis (Shi et al., 2021), or pyroptosis (Wu et al., 2021), could form gene signatures to predict the prognosis of patients with THCA. Cuproptosis is a new form of RCD, first reported by Tsvetkov et al., regulated by copper and different from other known regulatory mechanisms of cell death (Tsvetkov et al., 2022). Copper ions directly affect the tricarboxylic acid cycle pathway, leading to the loss of iron-sulfur cluster proteins, thereby triggering cell death (Tsvetkov et al., 2022). Currently, 10 regulatory genes related explicitly to copper death metabolic pathway have been identified, including 7 positive regulatory genes FDX1, LIAS, LIPT1, DLD, DLAT, PDHA1, and PDHB, and 3 negative regulatory genes MTF, GLS, and CDKN2A. Copper ion carriers such as disulfiram and elesclomol can function as therapeutic agents in cancer by inducing copper toxicity (Kirshner et al., 2008; Tsvetkov et al., 2019), representing a new approach to cue cancers by the unique function of copper. Up to now, the role of cuproptosis and the prognostic values of cuproptosis regulators remain unknown in THCA.

In the present study, we systematically analyzed the underlying biological function and prognostic value of cuproptosis regulators in THCA. We constructed a novel prognostic signature based on cuproptosis regulator FDX1 and cuproptosis inducer elesclomol to predict progression-free survival (PFS) of THCA patients. Furthermore, we explored the relationship between the cuproptosis-related score (RS) and enrichment score of biological pathways and immune cell infiltration levels. Eventually, we verified the cellular biological functions of FDX1 by knockdown of its expression and explored the regulation relationship with genes involved in the cuproptosis signature in THCA.

## 2 Materials and methods

### 2.1 Data preprocessing

We obtained the clinical information and raw fragment per kilobase (FPKM) values of THCA in The Cancer Genome Atlas (TCGA) and the Genotype-Tissue Expression (GTEx) datasets from the UCSC XENA database. We directly downloaded the series matrix files of the Affymetrix microarray profiles for GSE29265, GSE33630, GSE35570, and GSE60542 from the Gene Expression Omnibus (GEO) database. All the information about the public datasets is available in Supplementary Table S1. We used the “Combat” algorithm from the R package “sva” to eliminate the batch effects from different GEO datasets (Johnson et al., 2007), and we used the principal component analysis (PCA) to validate the impact of data normalization further. We named the aggregated four GEO datasets as GEO merge data groups for further research.

### 2.2 Single-sample gene set enrichment analysis (ssGSEA)

For biological function enrichment analysis, we employed a non-parametric and unsupervised method, ssGSEA, to estimate the activation of specific pathways based on gene signature “c5.all.v6.2.symbols” from the MSigDB database (Subramanian et al., 2005). Similarly, the relative abundance of immune cell infiltration levels in the tumor microenvironment (TME) was also conducted using the ssGSEA algorithm based on immune cell signature (Supplementary Table S2) (Bindea et al., 2013). Furthermore, the Database for Annotation, Visualization and Integrated Discovery (DAVID) v6.8 (Huang et al., 2009) was also employed to conduct GO analysis, consisting of biological processes, cellular component, molecular function, and the Kyoto Encyclopedia of Genes and Genomes (KEGG) pathways analysis.

### 2.3 Weighted gene co-expression network analysis (WGCNA)

To explore the underlying biological function of cuproptosis regulators in THCA, we clustered the THCA samples into several modules based on the expression patterns. Firstly, we used sample clustering to detect outliers in TCGA and GEO merge sets. The soft thresholding power was set as 9 for subsequent co-expression module establishment in two datasets. Module–trait associations were applied to set up a relationship between modules and the expression of cuproptosis. The functional enrichment analysis was conducted for GO and KEGG analysis for each expression module, respectively. All the intersection analyses were performed online, and the WGCNA algorithm (Langfelder and Horvath, 2008) was screened using R.

### 2.4 Establishment of the cuproptosis-related score (RS)

Genes were first filtered by correlation with both FDX1 expression and the predicted IC50 values of the cuproptosis inducer elesclomol in TCGA and GEO cohorts (|r| > 0.3, P < 0.05). Only genes satisfying these criteria in both datasets were retained and defined as candidate cuproptosis-related genes. These intersected genes were subsequently subjected to univariate Cox regression analysis, and genes with P < 0.05 were selected for feature selection. A Sliding Windows Sequential Forward Feature Selection (SWSFS) framework was then implemented using a Ranger-based random survival forest model (Zhang et al., 2019). Briefly, features were sequentially added in a forward manner, and at each step, model performance was evaluated using the out-of-bag (OOB) error as an internal cross-validation metric. The Ranger model was constructed with 1,000 trees, log-rank splitting rules, and a minimum node size of 15. The number of features yielding the lowest OOB error was considered optimal. Based on this procedure, six genes were selected to construct the final cuproptosis-related score (RS). The RS was calculated as: RS = Σ (Coefᵢ × Expᵢ), where Coefᵢ represents the regression coefficient and Expᵢ denotes the expression level of each gene. The median RS was used as the cutoff to stratify patients into high- and low-score groups. The cuproptosis RS could be calculated using the formula: cuproptosis RS = Σ (Coef i × Exp i), where i is the members involved in the gene signature. We performed stratification analysis to test whether the cuproptosis RS was an independent prognostic factor in THCA. Based on the members involved in cuproptosis RS, we built a prognostic nomogram using the “rms” R package to predict 1- and 3- years PFS of THCA, and the predictive accuracy of this nomogram was assessed using the calibration curve.

### 2.5 Cell culture

Normal thyroid cell line, Nthy-ori 3-1, and PTC cell lines, TPC-1 and BCPAP, were purchased from the National Collection of Authenticated Cell Cultures (Shanghai, China). All cell lines are maintained in RPMI1640 media supplemented with 10% FBS and 1% ampicillin/streptomycin and cultured at 5% CO_2_.

### 2.6 Transfection and real-time quantitative PCR (qRT-PCR) analysis

Small interfering RNA was purchased from GenePharma (Suzhou, China) and transfected using Lipofectamine 3000 (Invitrogen/Thermo Fisher Scientific). Total RNAs from cells were extracted using the Tiangen DNA kit (Tiangen Biotech, Beijing, China) and measured total RNA by SYBR Green One-Step qRT-PCR kit (Invitrogen, 11736059). The specific details of primers and siRNA sequences in this study are shown in Supplementary Table S3.

### 2.7 Cell counting kit-8 and colony formation assays

Approximately 1000 cells were seeded into six-well plates in triplicate and incubated for 5 days. Then cells were washed with PBS and fixed with 4% paraformaldehyde for 15 min, followed by 30 min incubation with 0.1% crystal violet. After enough washing by PBS, cell colonies containing more than 50 cells were counted and photographed. Cells were plated in 96-well plates (2000 cells/well), and the cell counting kit-8 (CCK-8) was performed by the manufacturer’s protocol (Biosharp, China).

### 2.8 Statistical analysis

The comparison of different groups was estimated using the Mann–Whitney U test (Wilcoxon rank-sum test). Spearman’s correlation analysis was performed to calculate the correlation coefficient between the two factors. Receiver operator characteristic curve (ROC) analysis was used to calculate the elements’ area under curve (AUC) value. The optimal cutoff point for a factor was determined using the “survminer” R package, and the “surv-cutpoint” function was used to repeat all potential cutoff points to obtain the best separation groups. Survival curves for prognostic analysis were employed using the Kaplan-Meier method, and significant differences were determined using the log-rank test. They represent immunohistochemical (IHC) images of cuproptosis regulators that were downloaded from the Human Protein Atlas (HPA) (https://www.proteinatlas.org). To predict the half-maximal inhibitory concentration (IC50) of a cuproptosis inducer, elesclomol, in a single sample, the Genomics of Drug Sensitivity in Cancer (GDSC) (https://www.cancerrxgene.org/) was used by R package “pRRophetic” following the instructions described previously (Yang et al., 2013). The Tumor Immune Estimation Resource (TIMER) analysis was conducted and visualized in Sangerbox database (http://sangerbox.com/). The asterisks represent the statistical P-value (*P < 0.05; **P < 0.01; ***P < 0.001).

## 3 Results

### 3.1 Comprehensive analysis of cuproptosis regulators in THCA

**FIGURE 1:**
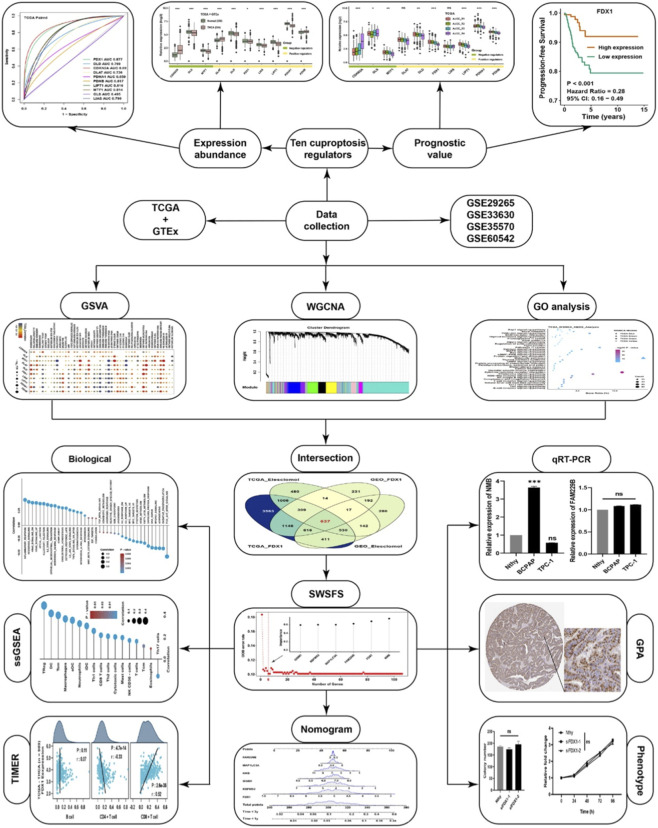
The flow chart of this study.

**FIGURE 2:**
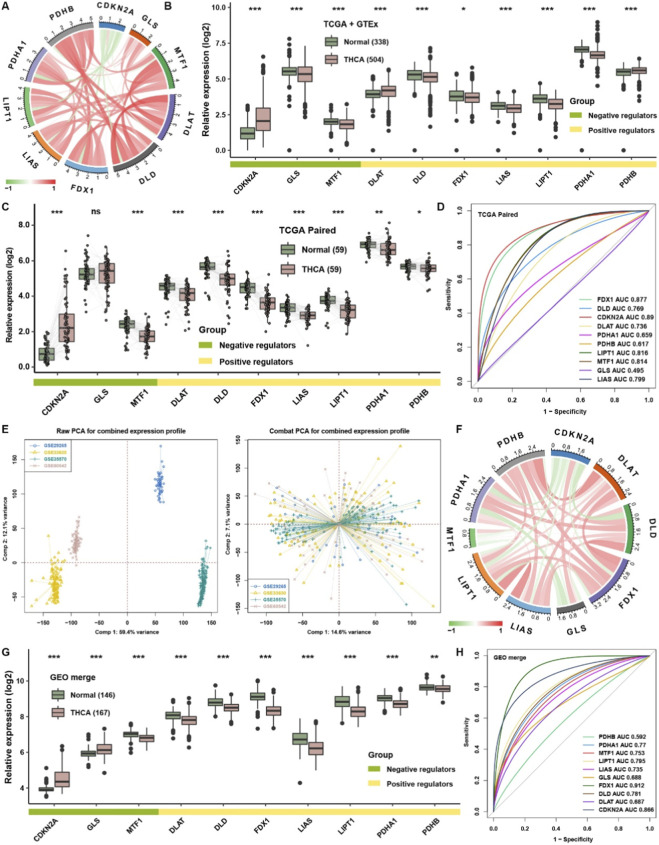
The expression level of cuproptosis regulators in THCA. **(A)** Correlation analysis among the cuproptosis regulators in TCGA. The color of the line represents the positive and negative correlation. The thickness of the line represents the level of the correlation. **(B)** Relative expression of cuproptosis regulators in TCGA and GTEx datasets. **(C)** Relative expression of cuproptosis regulators in TCGA paired samples. **(D)** ROC analysis for cuproptosis regulators between normal and THCA tissues in TCGA. **(E)** Principal component analysis (PCA) of the transcriptome profiles in distinct and combined GEO cohorts. **(F)** Correlation analysis among the cuproptosis regulators in GEO cohorts. **(G)** Relative expression of cuproptosis regulators in GEO cohorts. **(H)** ROC analysis for cuproptosis regulators between normal and THCA tissues in GEO cohorts. The asterisks represent the statistical p-value (*p < 0.05, **p < 0.01, and ***p < 0.001. ns, no significance).

**FIGURE 3:**
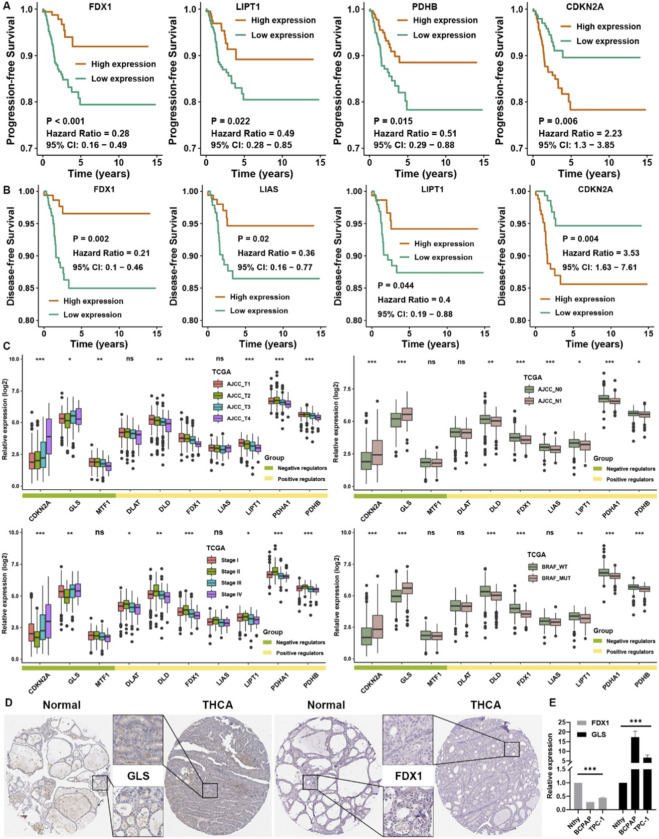
Prognostic value of cuproptosis regulators in THCA. **(A)** Survival analysis for cuproptosis regulators with progression-free survival data. **(B)** Survival analysis for cuproptosis regulators with disease-free survival data. **(C)** The relative expression level of cuproptosis regulators in different clinical groups. **(D)** The representative image of FDX1 and GLS in normal and THCA tissues from the HPA database. **(E)** The relative expression level of FDX1 and GLS in normal and THCA cell lines by qRT-PCR. The asterisks represent the statistical p-value (*p < 0.05, **p < 0.01, and ***p < 0.001. ns, no significance).

The main flowchart is shown in Figure 1. To explore the intrinsic relationship among the cuproptosis regulators in THCA, correlation analysis was first displayed among ten cuproptosis regulators in the TCGA cohort (Figure 2A). CDKN2A, a cuproptosis negative regulator, was negatively correlated with other cuproptosis positive regulators, indicating that a competitive relationship existed among the cuproptosis regulators. To elucidate the importance of cuproptosis regulators in THCA, differential analysis was conducted in GTEx and TCGA datasets. Most cuproptosis positive regulators, such as DLD, FDX1, LIAS, LIPT1, and PDHA1, were significantly lower expressed in THCA than in normal thyroid tissues (Figure 2B). Furthermore, the matched-pair analysis showed that all cuproptosis positive regulators were substantially lower expressed in THCA, while CDKN2A was observably higher in THCA than in normal THCA tissues (Figure 2C). ROC analysis further elucidated that the expression of cuproptosis regulators could well reflect the differences between the THCA and para-carcinoma tissues, the AUC of most of them was more than 0.7 (Figure 2D). For validation, we combined four GEO datasets as a whole cohort. PCA showed that the normalization effects were pretty good (Figure 2E). Correlation analysis showed that three cuproptosis negative regulators, CDKN2A, MTF1, and GLS, were negatively correlated with cuproptosis positive regulators (Figure 2F), which was consistent with the regulatory patterns of cuproptosis. Differentially expressed analysis further confirmed that all cuproptosis positive regulators were lower in THCA tissues, while two cuproptosis negative regulators, CDKN2A and GLS, were highly expressed in THCA tissues (Figure 2G). ROC analysis confirmed again that FDX1 and CDKN2A were representative cuproptosis regulators in THCA, with AUC >0.8 (Figure 2H). Survival analysis showed that THCA patients with higher expression of FDX1, LIPT1, and PDHB owned better progression-free survival rates (Figure 3A), and THCA patients with higher expression of FDX1, LIAS, LIPT1 showed better disease-free survival rates (Figure 3B). The higher expression of CDKN2A simultaneously showed worse progression-free and disease-free survival probability in THCA. From the perspective of clinical traits, we found that CDKN2A and GLS were higher expressed in T4, N1, stage IV, and BRAF mutation groups. At the same time, DLD, FDX1, LIPT1, PDHA1, and PDHB were significantly lower expressed in T4, N1, stage IV, and BRAF mutation groups (Figure 3C). We validated again at the protein level with the help of an IHC image from the HPA database. GLS was higher in THCA tissues, and FDX1 was negative in THCA tissues but positive in normal thyroid tissues (Figure 3D). Moreover, we could see that DLD, LIAS, and LIPT1 were higher in normal thyroid tissues, CDKN2A was significantly higher in THCA, and others showed no apparent differences by qualitative analysis (Supplementary Figure S1).

### 3.2 Systematically functional analysis of cuproptosis regulators in THCA

**FIGURE 4:**
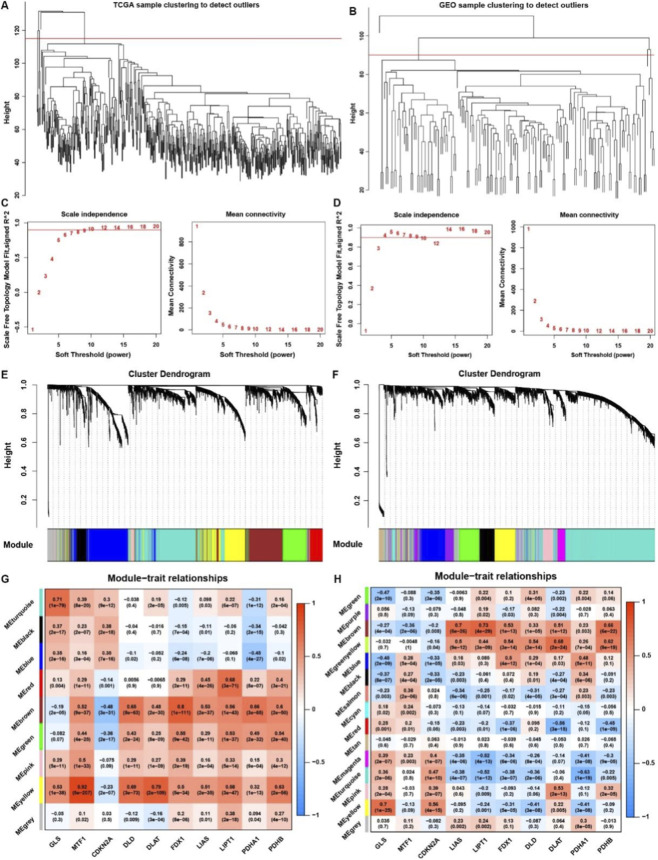
Construction steps of WGCNA. **(A,B)** Sample clustering to detect outliers in TCGA and GEO datasets. **(C,D)** Analysis of the network topology for various soft thresholding powers in TCGA and GEO datasets. The powers were both set as 9 for further research. **(E,F)** Clustering dendrograms with dissimilarity based on the topological overlap and the assigned module colors in TCGA and GEO datasets. **(G,H)** Module–trait relationships. Each row corresponds to a module eigengene, each column corresponds to a cuproptosis, and each cell consists of the corresponding correlation and P-value, color-coded by correlated according to the color legend.

**FIGURE 5:**
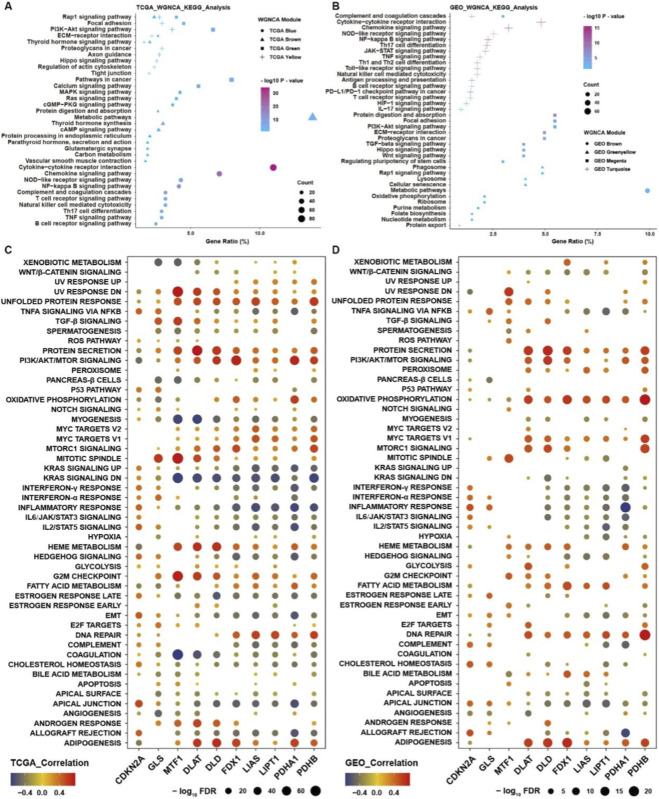
Biological function analysis of cuproptosis regulators in THCA. **(A)** KEGG analysis of genes involved in blue, brown, green, and yellow modules from WGCNA in TCGA. **(B)** KEGG analysis of genes involved in brown, green-yellow, magenta, and turquoise modules from WGCNA in GEO. **(C)** Correlation analysis between the expression of cuproptosis regulators and GSVA enrichment scores in TCGA. **(D)** Correlation analysis between the expression of cuproptosis regulators and GSVA enrichment scores in GEO.

For systematically analyzing the biological function of cuproptosis regulators in THCA, we employed a weighted gene co-expression network analysis (WGCNA) to construct a co-expression network. After removing the outlier samples, we analyzed the protein-coding RNAs with WGCNA to identify the modules containing highly correlated genes in TCGA and GEO merge datasets (Figures 4A,B). A soft threshold (β = 9) was used to guarantee a scale‐free network (Figures 4C,D), which identified eight modules and fourteen modules, respectively, from TCGA and GEO merge datasets (Figures 4E,F). Among these modules in the TCGA cohort, the blue module was significantly negatively correlated with cuproptosis positive regulators and positively correlated with cuproptosis negative regulators, while yellow, green, and brown modules were on the contrary (Figure 4G). As for the GEO merge cohort, brown, green-yellow, magenta and turquoise modules were also highly associated with cuproptosis regulators (Figure 4H). KEGG analysis showed that genes involved in the blue module were mainly enriched in cytokine-cytokine receptor interaction and chemokine signaling pathways, indicating that cuproptosis positive regulators might be highly negatively associated with immune-related pathways, such as T, B cells receptor and NK, TH17 cells differentiation pathways (Figure 5A). As for the other three modules positively correlated with cuproptosis positive regulators, Rap1, PI3K-AKT, Hippo, and MAPK signaling pathways associated with tumorigenesis and progression processes were highly enriched (Figure 5A). KEGG analysis in the GEO merge cohort verified the results of enrichment analysis from TCGA (Figure 5B), indicating that cuproptosis regulators might be involved in initiating and progressing tumorigenesis in THCA. For further research, we employed GSVA for validation in TCGA and GEO merge cohorts, respectively (Figures 5C,D). We found that most cuproptosis positive regulators positively correlated with protein secretion, oxidative phosphorylation, MYC, MTORC1, DNA repair, and adipogenesis while negatively correlated with immune-related pathways, such as interferon response and inflammatory pathways.

### 3.3 Construction of cuproptosis-related score for THCA

**FIGURE 6:**
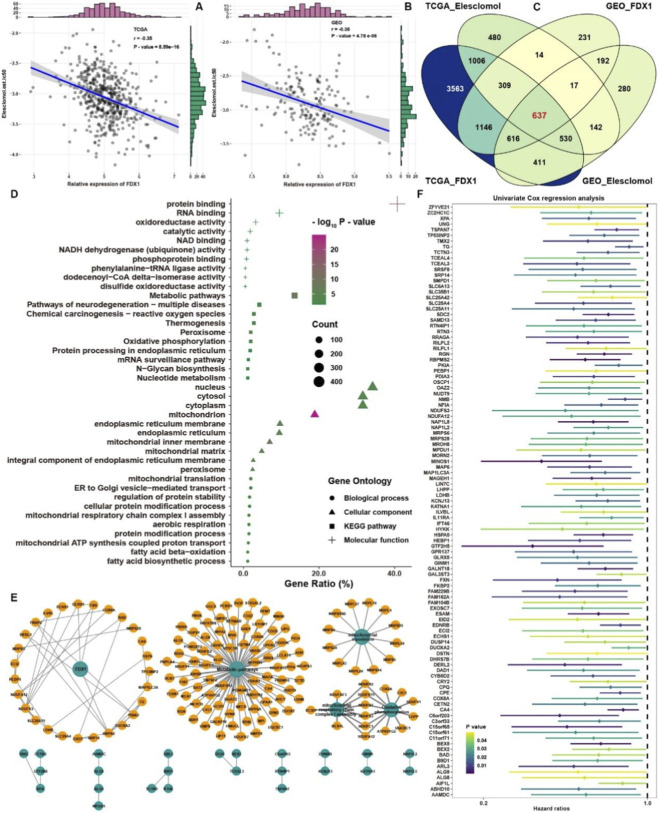
Identification of cuproptosis-related genes. **(A)** Correlation analysis between the expression of FDX1 and prediction IC50 of elesclomol in TCGA. **(B)** Correlation analysis between the expression of FDX1 and prediction IC50 of elesclomol in GEO. **(C)** The intersection of genes satisfied with criterion in TCGA and GEO. **(D)** GO and KEGG analysis of cuproptosis-related genes. **(E)** Protein-protein interaction network and function enrichment network of cuproptosis-related genes. **(F)** Univariate COX analysis of cuproptosis-related genes in TGCA with PFS data.

As FDX1 exhibited the most robust statistical associations among the ten cuproptosis-related genes, the above analyses highlight its potential value as a prognostic biomarker in THCA rather than implying a definitive functional dominance. We next chose FDX1 as a core analytic target. Correlation analysis showed that the expression level of FDX1 was significantly negatively correlated with the predicted IC50 of elesclomol, a well-known inducer of cuproptosis, by GDSC analysis in TCGA and GEO merge cohorts respectively (Figures 6A,B). Correlation analysis was conducted in TCGA and GEO merge cohorts, respectively, among the expression of protein-coding RNAs and the expression of FDX1, and the predicted IC50 of elesclomol. After the intersection among these groups, 637 intersected genes were selected as cuproptosis-related genes for further analysis (Figure 6C). These genes were satisfied with the criterion of | correlation coefficients | > 0.3 and P–value <0.05 (Supplementary Table S4). GO analysis showed that most of these genes were located at mitochondrial and associated with cell metabolism processes. KEGG analysis showed that reactive oxygen species and oxidative phosphorylation pathways were highly enriched (Figure 6D). The STRING database depicted the specific protein interaction relationship among these genes, and the correlation networks between genes and pathways were also shown in Figure 6E. The metabolic pathways and mitochondrial-related phenotypes were mainly involved here. Then, univariate COX analysis was conducted for these genes to explore the prognostic values for THCA (Figure 6F; Supplementary Table S5). With the criterion of P–value <0.05, 105 genes were selected for further analysis.

**FIGURE 7:**
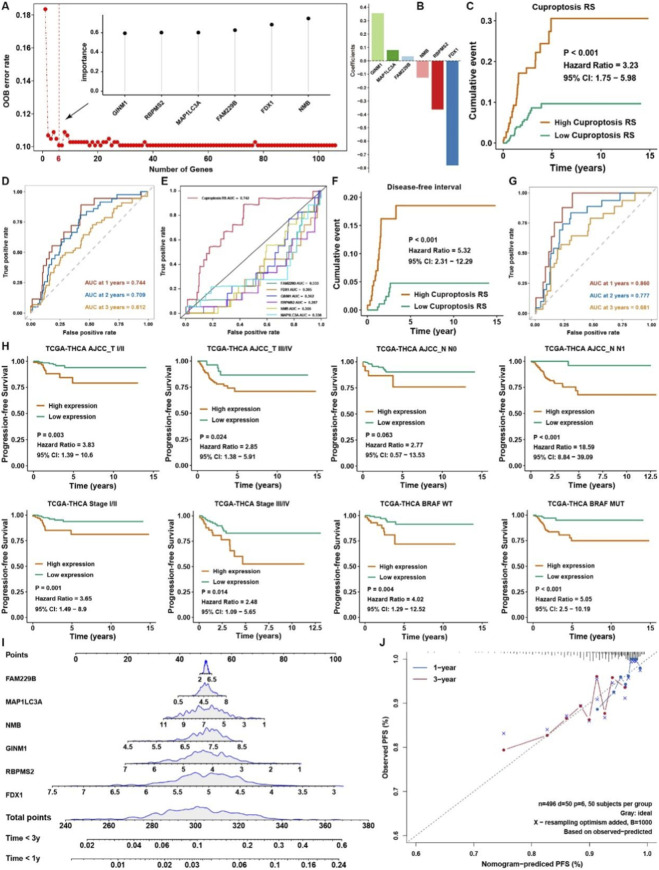
Construction of cuproptosis RS signature for THCA. **(A)** The relative importance of each cuproptosis-related gene was calculated by random survival forest analysis, and the 6 most critical genes are shown. **(B)** Coefficients of 6 genes involved in the cuproptosis RS. **(C)** Cumulative incidence of PFS in high and low cuproptosis RS groups. **(D)** The time-dependent AUC values of the cuproptosis RS for predicting 1-, 2- and 3-year PFS in TCGA. **(E)** ROC analysis of cuproptosis RS and its members for THCA progression state. **(F)** Cumulative incidence of DFS in high and low cuproptosis RS groups. **(G)** The time-dependent AUC values of the cuproptosis RS for predicting 1-, 2- and 3-year DFS in TCGA. **(H)** Survival analysis of the PFS in THCA patients with different clinical traits stratified by the cuproptosis RS. **(I)** Nomogram to predict the 1- and 3-year PFS of THCA patients. **(J)** Calibration curve for the comprehensive survival nomogram model. The dashed diagonal line represents the ideal situation, and the blue and red lines represent the 1- and 3-year observed nomograms, respectively.

Then, as shown in Figure 7A, we employed Ranger, a weighted version of random forest based on the SWSFS algorithm, to evaluate the importance of each cuproptosis-related gene. After processing with the SWSFS algorithm, the results showed that when the number of genes was 6, the out-of-bag (OOB) error rate was the lowest, indicating that its predictive ability was the strongest. Thus, the importance of these 6 genes is in the upper right of Figure 7A. And the coefficients of these 6 genes were shown in Figure 7B; Supplementary Table S6, and the cuproptosis-related score (RS) = −0.78044 * FDX1 - 0.36267 * RBPMS2 - 0.12573 * NMB +0.08012 * MAP1LC3A+ 0.35546 * GINM1 + 0.03373 * FAM229B. Survival analysis showed that THCA samples in the high cuproptosis RS group possessed a worse survival rate than those in low cuproptosis RS (Figure 7C). Moreover, the time-dependent AUC values of the cuproptosis RS for predicting 1-, 2- and 3-year progression-free survival rates in THCA exceeded 0.6 (Figure 7D). Compared with a single gene involved in cuproptosis RS as an aspect of PFS prediction, cuproptosis RS showed excellent predicted efficacy than all of them (Figure 7E). As for the DFS rate, the cuproptosis RS could also predict well (Figure 7F), and the AUC values exceeded 0.68 for the prediction of 1-, 2- and 3-year disease-free survival rates in THCA (Figure 7G). Stratified analysis showed that a high cuproptosis RS was correlated with dramatically worse PFS, regardless of whether the patient exhibited early- or advanced-stage THCA (Figure 7H). Likewise, we also found that regardless of THCA belonging to BRAF wild-type (WT) or BRAF mutation (MUT), the cuproptosis RS provided statistically significant PFS stratification (Figure 7H). To further facilitate the clinical use of the cuproptosis RS, a nomogram capable of predicting the 1- or 3-year survival probability of THCA patients was finally constructed (Figure 7I). The calibration curves at 1 and 3 years indicated good consistency between the prediction by the nomogram and actual PFS (Figure 7J).

### 3.4 Identification of cuproptosis RS-related biological characteristics

**FIGURE 8:**
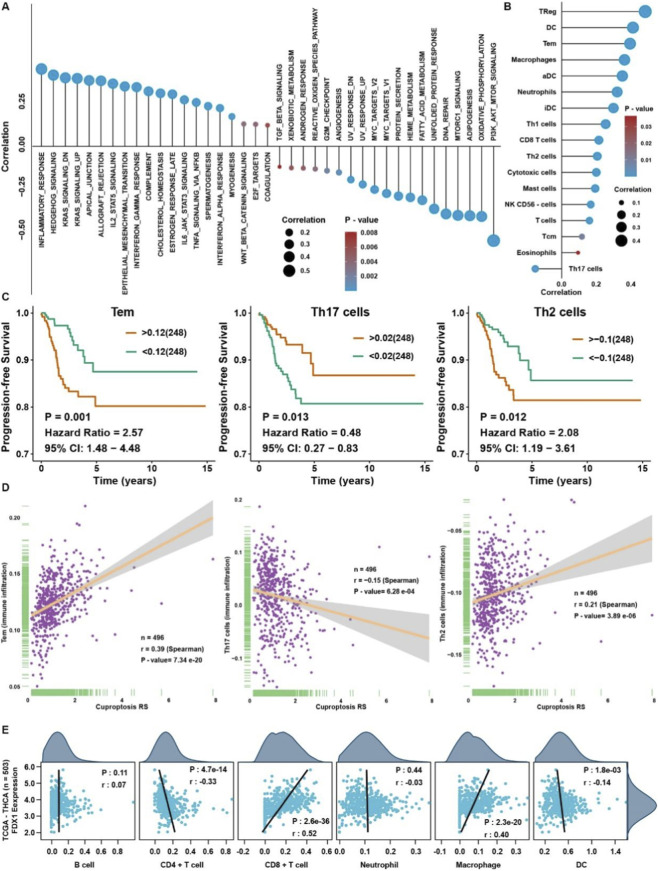
Relationship among cuproptosis RS and FDX1 with immune cell infiltration. **(A)** Correlation analysis between the cuproptosis RS and GSVA enrichment scores. **(B)** Correlation analysis between the cuproptosis RS and immune cell infiltration levels. **(C)** Survival analysis for immune cell infiltration levels. **(D)** Correlation analysis between the cuproptosis RS and Tem, Th17, and Th2 cells. **(E)** Correlation analysis between the cuproptosis RS and the results of TIMER in THCA.

As the cuproptosis RS displayed potent prognosis prediction efficacy, we wonder if there was any underlying biological function in the process of THCA. Firstly, a correlation analysis was conducted between the GSVA enrichment scores and cuproptosis RS (Figure 8A). We found that PI3K/AKT, oxidative phosphorylation, adipogenesis, MTORC1, and DNA repair pathways were significantly negatively correlated with cuproptosis RS. In contrast, inflammatory, Hedgehog, and KRAS signaling pathways are positively associated with cuproptosis RS. It indicated that the high cuproptosis RS group displayed higher growth and inflammatory signaling, but the low cuproptosis RS group possessed higher oxidative stress levels.

Because cuproptosis RS is strongly associated with immune-related pathways, immune cell infiltration levels were assessed by ssGSEA in THCA. We found that most immune cells correlated with cuproptosis RS (Figure 8B). Survival analysis showed that effector memory T cell (Tem) and Th2 cell were a risk factor for THCA, but Th17 cell was a protective factor for THCA (Figure 8C). Correlation analysis showed that cuproptosis RS was positively correlated with Tem and Th2 cells but negatively correlated with Th17 cells (Figure 8D). To validate the relationship between FDX1 and immune cells infiltration level, TIMER showed that FDX1 was significantly associated with CD8^+^ T cells and macrophages (Figure 8E).

### 3.5 Expression validation for genes in cuproptosis RS and biological function exploration of FDX1 in THCA

**FIGURE 9:**
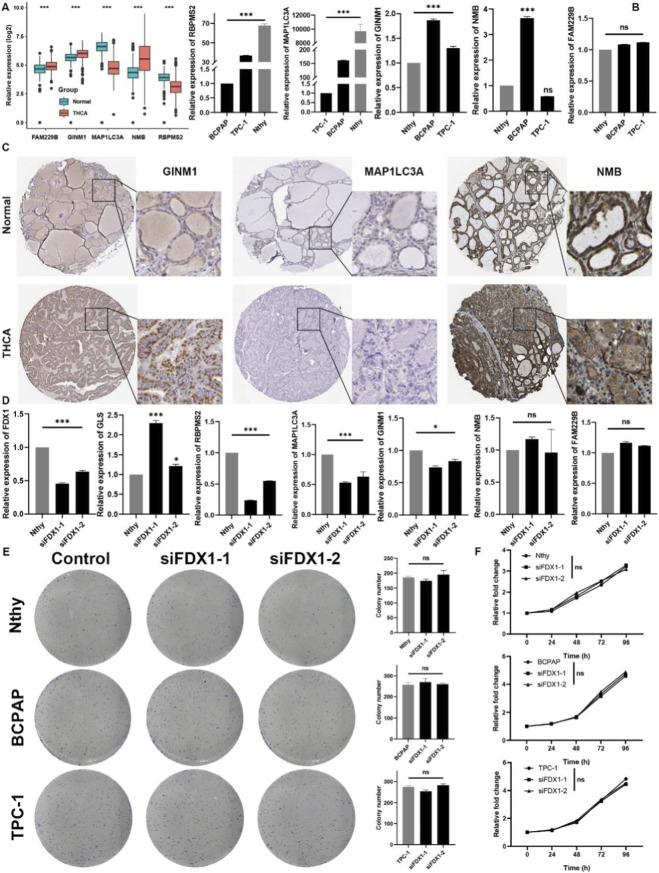
Expression validation for genes in cuproptosis RS and biological function exploration of FDX1 in THCA. **(A)** Expression levels of cuproptosis RS members between normal and THCA tissues in TCGA, **(B)** Experimental verification of the expression levels of cuproptosis RS members between normal cell line and THCA cell lines through qRT-PCR. **(C)** Representative images of cuproptosis RS members from the HPA database. **(D)** Expression levels of cuproptosis RS members after downregulated of FDX1 in Nthy cell line by qRT-PCR. **(E)** Colony forming assays in normal and THCA cell lines after down-regulated of FDX1. **(F)** CCK-8 assays of normal and THCA cell lines after downregulated of FDX1. The asterisks represent the statistical p-value (*p < 0.05 and ***p < 0.001. ns, no significance).

To further elucidate the importance of genes involved in cuproptosis RS signature, we found that FAM229B, GINM1, and NMB were significantly higher expressed in THCA. At the same time, the expression of MAP1LC3A and RBPMS2 were markedly lower in THCA (Figure 9A). Using qRT-PCR, we found that the expression level of MAP1LC3A and RBPMS2 were dramatically upregulated in a normal thyroid cell line in comparison with. Still, the expression level of GINM1 was significantly higher in THCA cell lines (Figure 9B). The expression level of NMB and FAM229B was uncertain between THCA and the normal cell line. With the help of HPA, we confirmed that the expression of GINM1 was remarkably higher in THCA tissues, and MAP1LC3A was more elevated in normal tissues (Figure 9C). However, the expression level of NMB was both strong in THCA or normal thyroid tissues, and the IHC images of RBPMS2 and FAM229B remained uncertain. To investigate whether the biological function of FDX1 is related to the growth of THCA, the knockdown of FDX1 in cell lines was conducted using small interference RNA. Through qPCR, we found that the siRNAs significantly inhibited the expression of FDX1 (Figure 9D). Surprisingly, we found that the expression level of cuproptosis negative regulator GLS was elevated, while MAP1LC3A, RBPMS2, and GINM1 were downregulated (Figure 9D). Unexpectedly, the results of the colony-formation experiment and CCK-8 assays suggested that the knockdown of FDX1 could not affect the proliferation ability of thyroid cells (Figures 9E,F).

## 4 Discussion

In this study, we first systematically analyzed the cuproptosis regulators in thyroid cancer from the perspective of expression level and prognostic values. As we all know, thyroid cancer with a high incidence was not sensitive to many treatments, including chemo- or radiotherapy. Exploring novel alternative therapeutic methods in cell death mechanisms was innovative and efficient. We found that most cuproptosis positive regulators were downregulated in cancer tissues than in normal tissues, while two cuproptosis negative regulators, GLS and CDKN2A, were on the contrary. These cuproptosis regulators have previously been reported to play essential roles in tumors. FDX1 has been reported to play an important role in metabolism and is closely related to mitochondrial cytochrome (Sheftel et al., 2010; Strushkevich et al., 2011; Shi et al., 2012). Here, we found that FDX1 was the most significant cuproptosis regulator between THCA and normal tissues with AUC >0.9 and prognostic value for PFS and DFS with hazard ratio <1. Interestingly, GLS was negatively correlated with FDX1 and displayed a relationship with the advanced tumor stage. Using qRT-PCR and correlation analysis, we speculated competitive connection might have existed between FDX1 and GLS. GLS is a crucial enzyme in glutamine metabolism as it catalyzes the transformation of glutamine to glutamate, which is further converted into produce α-ketoglutarate (Song et al., 2018). It has been reported to upregulate cell metabolism for tumor growth and is considered to be a potential therapeutic target for cancer treatment (Xiang et al., 2015; Kitayama et al., 2017).

Given better overall survival of THCA, we are more concerned about progression- or disease-free survival. Although there has been a lot of work on prognostic models for THCA, few have focused on PFS or DFS mainly but mostly on OS. The signature used to construct the model are also not covered cuproptosis-related genes. We screened for underlying cuproptosis-related genes through the expression of crucial regulator FDX1 and the prediction IC50 of cuproptosis inducer elesclomol, even intersecting genes, in two independent datasets. As for methodology, SWSFS was first used in THCA to construct a prognostic signature. The detailed stratified analysis and the nomogram construction illustrated the validity and accessibility of this cuproptosis signature. Unfortunately, as no additional independent cohort with compatible transcriptomic profiles and PFS/DFS endpoints was available, we did not add new datasets. Instead, we have explicitly acknowledged this limitation in the Results and Discussion sections and reframed the model as an exploratory, hypothesis-generating prognostic framework. The independent clinical cohorts for validation are lacking and necessary for the clinical trial in the future.

From the coefficients of the cuproptosis signature, it was apparent that FDX1 was the most significant contributor and RBPMS2 took second place. This indicated that FDX1 dominated the accuracy for the signature, and other members might be affected by the change of it. Results from the database and cell lines confirmed that MAP1LC3A and RBPMS2 were lower in THCA, and GINM1 was higher in THCA than in normal tissues. Interestingly, after the knockdown of FDX1, MAP1LC3A, RBPMS2, and GINM1 were downregulated in Nthy cells. This inferred that these genes might also play an essential part in the process of cuproptosis and might be regulated by FDX1. MAP1LC3A encodes a light chain subunit that can associate with either MAP1A or MAP1B, which are microtubule-associated proteins that mediate the physical interactions between microtubules and components of the cytoskeleton (Wang et al., 2019). The expression of MAP1LC3A was reported to be suppressed in many tumor cell lines, such as gastric cancer, esophageal squamous carcinoma, osteosarcoma, and glioma (Bai et al., 2012; Giatromanolaki et al., 2014). It has been indicated that the products of MAP1LC3A can serve as autophagic markers and show autophagic activity (Zhang et al., 2016). RBPMS2 is a member of the RNA recognition motif-containing protein family (Sagnol et al., 2014), and aberrant expression of RBPMS2 can be observed explicitly in gastrointestinal mesenchymal neoplasm and digestive myopathy syndrome (Notarnico et al., 2012; Hapkova et al., 2013). But the biological function of RBPMS2 remains unclear in the carcinogenesis of tumors. We observed higher expression levels for glycosylation of glycoprotein integral membrane 1 (GINM1) in THCA patients or cell lines. However, the role of this protein in cancer development or progression is not yet reported.

The above analyses highlight the statistical and prognostic relevance of FDX1 in THCA. However, these findings primarily support its role as a biomarker rather than establishing FDX1 as a direct functional effector in cuproptosis or tumor progression. This point has been reported before in lung Adenocarcinoma, but it has been verified to mediate the metabolism (Zhang et al., 2021). From the results of GSVA, FDX1 was mainly correlated with MTORCI1 and metabolism-related pathways. Several immune-related pathways, such as interferon and inflammatory pathways, were negatively correlated with the expression of FDX1. This finding urged us to explore the relationship between the FDX1 and the immune cell infiltration levels. Thus, we found that the FDX1 might positively correlate with CD8^+^ T and macrophage cells. In addition to tumor cell initiation, the microenvironment mechanisms act as characteristics of tumor progression and relapse and might guide the treatment regimen in PTC (Al-Abdallah et al., 2020; Stenman et al., 2021). The combined effects of immune cell infiltration and inducing cuproptosis in PTC should be considered in future studies. It should be emphasized that the role of FDX1 as a prognostic biomarker is not equivalent to its role as a functional effector. The current experimental data primarily demonstrate transcriptional associations and do not establish a causal mechanism linking FDX1 to cuproptosis or immune regulation in THCA.

## 5 Conclusion

In conclusion, we confirmed FDX1 as a core cuproptosis regulator in THCA, and we filtered cuproptosis-related genes using correlation analysis with the expression of FDX1 and the predicted IC50 of elesclomol. With the help of the machine learning method, a cuproptosis RS signature was constructed to predict PFS or DFS for THCA patients. In addition, stratified analysis and nomogram were employed to verify the validity and accessibility of the signature. Experimental analyses indicate that FDX1 expression is closely associated with the cuproptosis-related transcriptional landscape. However, its functional involvement in cuproptosis and tumor biology remains to be established by future mechanistic studies.

## Data Availability

The data that support the findings of this study are available from the corresponding author upon reasonable request.
