# Supplementary material for: Comprehensive analyses of biological function and tumor microenvironment with cuproptosis regulators and construction of a cuproptosis-related scoring system in thyroid cancer based on bioinformatics and experimental validation
Source: Front Genet. 2026 Feb 27;17:1735093. doi: 10.3389/fgene.2026.1735093 (PMC12981724; doi:10.3389/fgene.2026.1735093)

**Supplementary Figure 1.** Representative IHC images of cuproptosis regulators in normal and THCA tissues from HPA database.
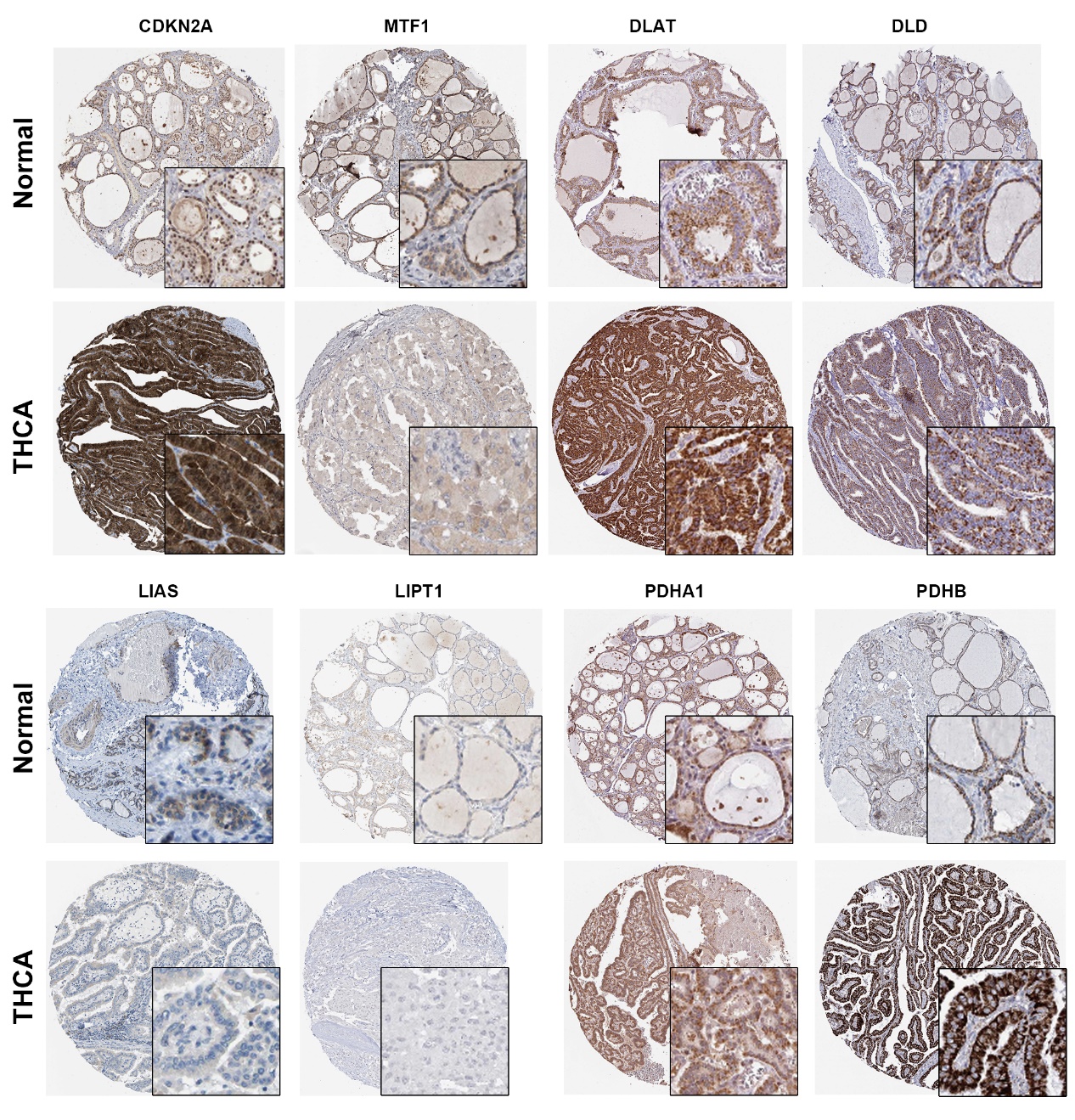

Supplement: Supplementary file 2 [file DataSheet1.docx]
